# 2,2′-Diiodo­azobenzene

**DOI:** 10.1107/S1600536812040718

**Published:** 2012-10-13

**Authors:** Philip J. W. Elder, Ignacio Vargas-Baca

**Affiliations:** a Department of Chemistry and Chemical Biology, McMaster University, 1280 Main, Street West, Hamilton, Ontario, Canada L8S 4M1

## Abstract

The mol­ecular structure of the title compound, C_12_H_8_I_2_N_2_ [systematic name: (*E*)-bis­(2-iodo­phen­yl)diazene], exhibits an essentially planar *trans* geometry [maximum deviation = 0.022 (4) Å] with the iodine atoms *ortho* to the azo bridge. In the crystal, offset π-stacking leads to the formation of columns along the *a* axis [closest C⋯C distance = 3.383 (4) Å].

## Related literature

For analogous 2,2′-dichloro­azobenzenes, see: Komeyama *et al.* (1973 ▶); Crispini *et al.* (1998 ▶). For the structure of a related *o*-halogenated azobenzene, see: Wragg *et al.* (2011 ▶).
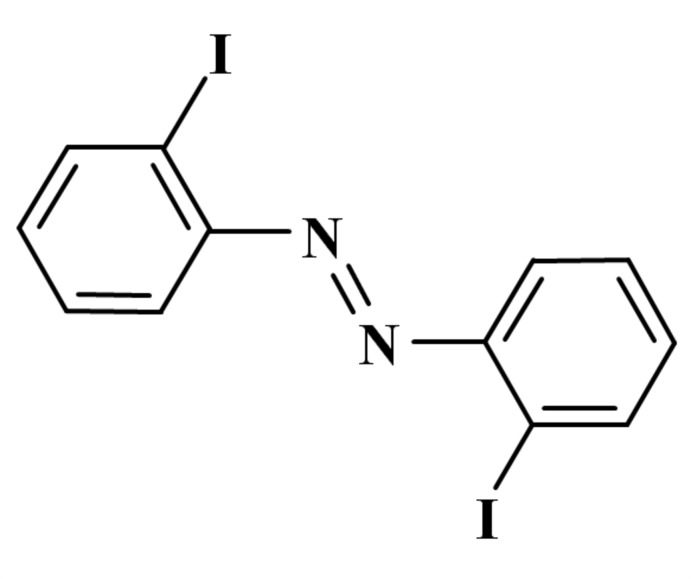


## Experimental

### 

#### Crystal data

C_12_H_8_I_2_N_2_
*M* *_r_* = 433.88
Monoclinic, 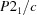

*a* = 4.6306 (3) Å
*b* = 18.1105 (12) Å
*c* = 15.3748 (10) Å
β = 98.532 (1)°
*V* = 1275.10 (14) Å^3^
*Z* = 4
Mo *K*α radiation
μ = 4.91 mm^−1^
*T* = 296 K
0.63 × 0.09 × 0.04 mm

#### Data collection

Bruker SMART CCD area-detector diffractometer
Absorption correction: analytical (*SADABS*; Sheldrick, 1996 ▶) *T*_min_ = 0.322, *T*_max_ = 0.873
16726 measured reflections
3186 independent reflections
2536 reflections with *I* > 2σ(*I*)
*R*_int_ = 0.027

#### Refinement

*R*[*F* ^2^ > 2σ(*F* ^2^)] = 0.027
*wR*(*F* ^2^) = 0.059
*S* = 1.03
3186 reflections
145 parameters
H-atom parameters constrained
Δρ_max_ = 0.56 e Å^−3^
Δρ_min_ = −0.56 e Å^−3^

### 

Data collection: *SMART* (Bruker, 2000 ▶); cell refinement: *SMART* (Bruker, 2000 ▶); data reduction: *SAINT*; program(s) used to solve structure: *SHELXTL* (Sheldrick, 2008 ▶); program(s) used to refine structure: *SHELXL97* (Sheldrick, 2008 ▶); molecular graphics: *Mercury* (Macrae *et al.*, 2006 ▶); software used to prepare material for publication: *SHELXTL*.

## Supplementary Material

Crystal structure: contains datablock(s) global, I. DOI: 10.1107/S1600536812040718/tk5154sup1.cif

Structure factors: contains datablock(s) I. DOI: 10.1107/S1600536812040718/tk5154Isup2.hkl

Supplementary material file. DOI: 10.1107/S1600536812040718/tk5154Isup3.cml

Additional supplementary materials:  crystallographic information; 3D view; checkCIF report

## Figures and Tables

**Fig. 1.**
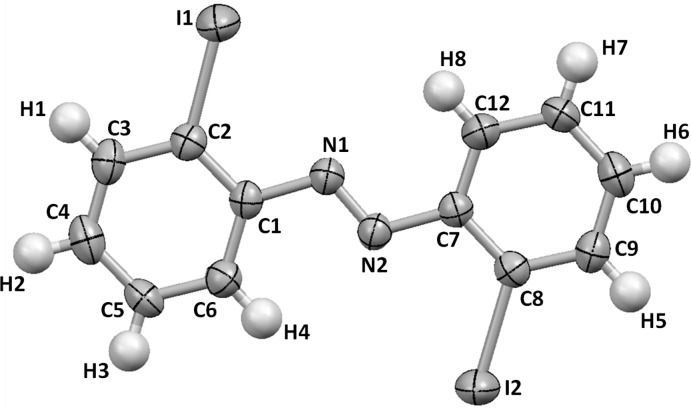
Perspective view of the crystal structure of 2,2'-diiodoazobenzene. Atoms are represented by their anisotropic displacement ellipsoids at 50% probability level. Hydrogen atoms are displayed as fixed-size spheres of 0.35 Å radius.

**Fig. 2.**
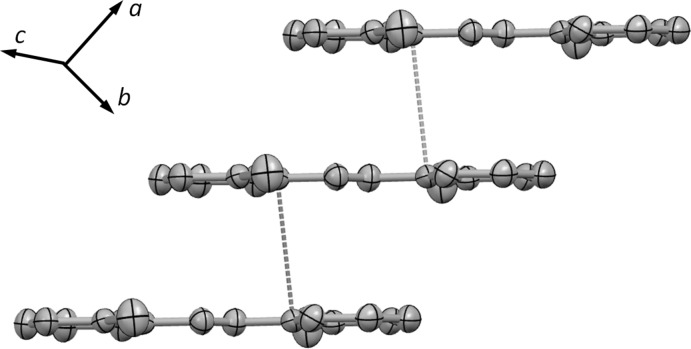
Intermolecular C1—C7* contacts (- - -) in the crystal of 2,2'-diiodoazobenzene. Hydrogen atoms are omitted for clarity.

**Fig. 3.**
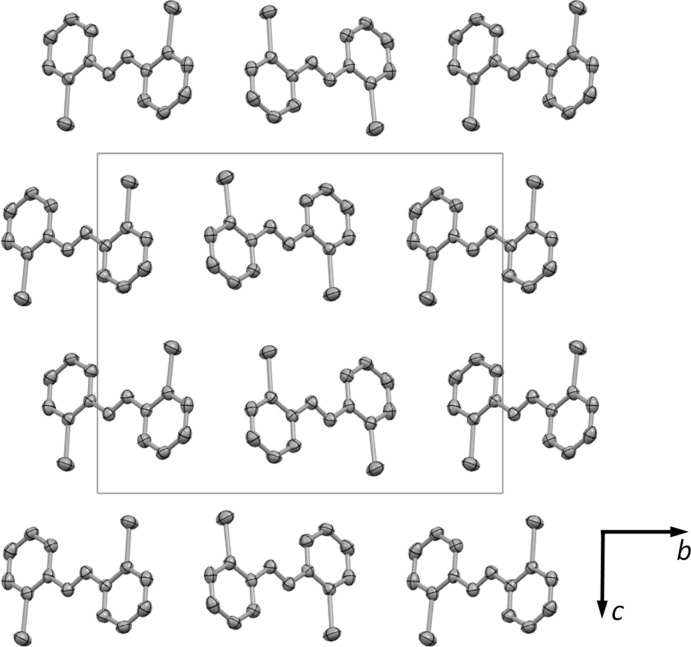
Packing diagram of 2,2'-diiodoazobenzene viewed along the a axis. Hydrogen atoms are omitted for clarity.

**Fig. 4.**
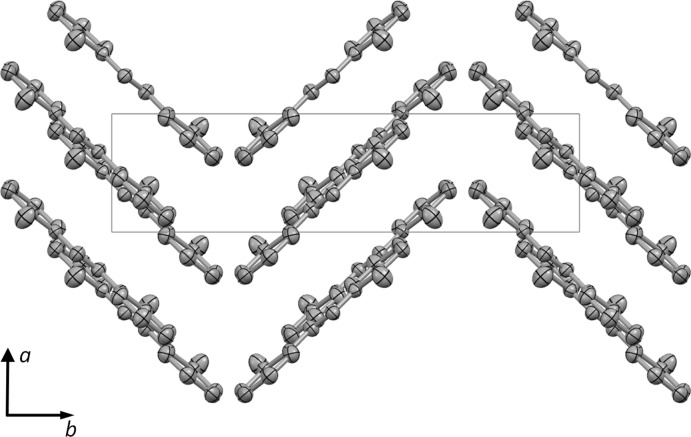
Packing diagram of 2,2'-diiodoazobenzene viewed along the c axis. Hydrogen atoms are omitted for clarity.

## supplementary crystallographic information

### Comment

The molecules of 2,2'-diiodoazobenzene exhibit a *trans* geometry with the iodine atoms in positions *ortho* to the azo bridge and opposite the N═N double bond (Fig. 1). The molecules are nearly planar, with the maximum deviation from the average plane being 0.022 (4) Å for atom I1. The aromatic rings of 2,2'-diiodoazobenzene are nearly co-planar with each other (interplanar angle = 0.08 (3)°) and with the azo bridge (N1—N2—C7—C12 = 0.5 (4)°; N2—N1—C1—C6 = -0.1 (4)°). These features are also observed in the structure of 2-iodoazobenzene (Wragg *et al.*, 2011). In contrast, the structures of dichloro analogues display parallel aromatic rings that are rotated from the plane of the azo bridge with N—N—C—C angles = 14.30 (6)° and -14.30 (6)° (Komeyama *et al.*, 1973), and 14.4 (3)° and -14.4 (1)° (Crispini *et al.*, 1998); the corresponding interplanar distances are 0.173 (1) and 0.351 (3) Å, respectively. Such structural differences are likely linked to the presence of intermolecular contacts in the structures of the iodo derivatives and their absence in the dichloro compounds. An offset π-stacking pattern (Fig. 2) allows significant overlap of adjacent molecules. The shortest intermolecular contact in 2,2'-diiodoazobenzene is between C1 and C7* (3.383 (4) Å, *cf.* sum of van der Waals radii = 3.40 Å; symmetry operation: 1+*x*, *y*, *z).* The stacking leads a columnar arrangement along *a* (Fig. 3). A herringbone pattern is observed perpendicular to the *c* axis (Fig. 4).

### Experimental

Azobenzene (0.184 g, 1.01 mmol) and mercury trifluoroacetate (0.43 g, 1.01 mmol) were combined with freshly distilled trifluoroacetic acid (0.13 mL) under a nitrogen atmosphere. The mixture was heated with stirring for 4 h at 68 °C, after which a concentrated solution of sodium chloride (0.345 g, 5.90 mmol) and sodium acetate (2.085 g, 14.7 mmol) was added and the entire sample was placed in an ultrasonic bath for 20 min. After decanting the solvent, a mixture of iodine (0.279 g, 1.10 mmol) in methanol was added. With time, orange crystals of 2,2'-diiodoazobenzene grew from the solution and were collected by filtration. Yield = 0.047 g, 10%.

### Refinement

Carbon-bound H-atoms were placed in calculated positions (C–H 0.93 Å) and were included in the refinement in the riding model approximation with *U*_iso_(H) set to 1.2*U*_eq_(C).

### Crystal data

**Table:**

C_12_H_8_I_2_N_2_	*F*(000) = 800
*M**_r_* = 433.88	*D*_x_ = 2.261 Mg m^−^^3^
Monoclinic, *P*2_1_/*c*	Mo *K*α radiation, λ = 0.71073 Å
Hall symbol: -P 2ybc	Cell parameters from 4767 reflections
*a* = 4.6306 (3) Å	θ = 2.6–24.6°
*b* = 18.1105 (12) Å	µ = 4.91 mm^−^^1^
*c* = 15.3748 (10) Å	*T* = 296 K
β = 98.532 (1)°	Rod, orange
*V* = 1275.10 (14) Å^3^	0.63 × 0.09 × 0.04 mm
*Z* = 4	

### Data collection

**Table:**

Bruker SMART CCD area-detector diffractometer	3186 independent reflections
Radiation source: fine-focus sealed tube	2536 reflections with *I* > 2σ(*I*)
Graphite monochromator	*R*_int_ = 0.027
φ and ω scans	θ_max_ = 28.4°, θ_min_ = 2.3°
Absorption correction: analytical (*SADABS*; Sheldrick, 1996)	*h* = −4→6
*T*_min_ = 0.322, *T*_max_ = 0.873	*k* = −24→22
16726 measured reflections	*l* = −20→18

### Refinement

**Table:**

Refinement on *F*^2^	Primary atom site location: structure-invariant direct methods
Least-squares matrix: full	Secondary atom site location: difference Fourier map
*R*[*F*^2^ > 2σ(*F*^2^)] = 0.027	Hydrogen site location: inferred from neighbouring sites
*wR*(*F*^2^) = 0.059	H-atom parameters constrained
*S* = 1.03	*w* = 1/[σ^2^(*F*_o_^2^) + (0.0243*P*)^2^ + 0.9205*P*] where *P* = (*F*_o_^2^ + 2*F*_c_^2^)/3
3186 reflections	(Δ/σ)_max_ = 0.001
145 parameters	Δρ_max_ = 0.56 e Å^−^^3^
0 restraints	Δρ_min_ = −0.56 e Å^−^^3^

### Special details

**Table:**

Experimental. Azobenzene (0.184 g, 1.01 mmol) and mercury trifluoroacetate (0.43 g, 1.01 mmol) were combined with freshly distilled trifluoroacetic acid (0.13 mL) under a nitrogen atmosphere. The mixture was heated with stirring during 4 h at 68°C, after which a concentrated solution of sodium chloride (0.345 g, 5.90 mmol) and sodium acetate (2.085 g, 14.7 mmol) was added and the entire sample was placed in an ultrasonic bath for 20 min. After decanting the solvent, a mixture of iodine (0.279 g, 1.10 mmol) in methanol was added. With time, crystals of 2,2'-diiodoazobenzene grew from the solution and were collected by filtration. Yield = 0.047 g, 10%.
Geometry. All s.u.'s (except the s.u. in the dihedral angle between two l.s. planes) are estimated using the full covariance matrix. The cell s.u.'s are taken into account individually in the estimation of s.u.'s in distances, angles and torsion angles; correlations between s.u.'s in cell parameters are only used when they are defined by crystal symmetry. An approximate (isotropic) treatment of cell s.u.'s is used for estimating s.u.'s involving l.s. planes.
Refinement. Refinement of *F*^2^ against ALL reflections. The weighted *R*-factor *wR* and goodness of fit *S* are based on *F*^2^, conventional *R*-factors *R* are based on *F*, with *F* set to zero for negative *F*^2^. The threshold expression of *F*^2^ > 2σ(*F*^2^) is used only for calculating *R*-factors(gt) *etc*. and is not relevant to the choice of reflections for refinement. *R*-factors based on *F*^2^ are statistically about twice as large as those based on *F*, and *R*- factors based on ALL data will be even larger.

### Fractional atomic coordinates and isotropic or equivalent isotropic displacement parameters (Å^2^)

**Table:**

	*x*	*y*	*z*	*U*_iso_*/*U*_eq_	
C1	1.0033 (6)	0.62044 (15)	0.74337 (19)	0.0362 (6)	
C2	1.1608 (6)	0.67355 (16)	0.7950 (2)	0.0391 (6)	
C3	1.3471 (7)	0.72094 (17)	0.7587 (2)	0.0469 (7)	
C4	1.3732 (7)	0.71515 (18)	0.6711 (2)	0.0511 (8)	
C5	1.2147 (7)	0.66291 (18)	0.6191 (2)	0.0447 (7)	
C6	1.0330 (7)	0.61521 (17)	0.6548 (2)	0.0440 (7)	
I1	1.12360 (6)	0.685129 (15)	0.927841 (16)	0.06511 (10)	
H1	1.4537	0.7564	0.7936	0.056*	
H2	1.4983	0.7466	0.6468	0.061*	
H3	1.2306	0.6599	0.5596	0.054*	
H4	0.9297	0.5794	0.6196	0.053*	
N1	0.8151 (5)	0.57362 (13)	0.78379 (16)	0.0409 (6)	
N2	0.6804 (5)	0.52810 (14)	0.73326 (16)	0.0400 (5)	
C7	0.4922 (6)	0.48055 (16)	0.77216 (18)	0.0366 (6)	
C8	0.3371 (6)	0.42818 (16)	0.71848 (19)	0.0380 (6)	
C9	0.1496 (6)	0.37976 (17)	0.7525 (2)	0.0451 (7)	
C10	0.1187 (7)	0.38397 (18)	0.8396 (2)	0.0511 (8)	
C11	0.2731 (7)	0.43536 (19)	0.8935 (2)	0.0494 (8)	
C12	0.4562 (7)	0.48416 (18)	0.8602 (2)	0.0480 (8)	
I2	0.37973 (5)	0.421268 (15)	0.585578 (15)	0.06002 (9)	
H5	0.0457	0.3447	0.7163	0.054*	
H6	−0.0076	0.3518	0.8624	0.061*	
H7	0.2537	0.4371	0.9528	0.059*	
H8	0.5561	0.5196	0.8967	0.058*	

### Atomic displacement parameters (Å^2^)

**Table:**

	*U*^11^	*U*^22^	*U*^33^	*U*^12^	*U*^13^	*U*^23^
C1	0.0328 (14)	0.0323 (15)	0.0429 (16)	0.0017 (12)	0.0041 (12)	0.0035 (12)
C2	0.0373 (15)	0.0366 (16)	0.0435 (16)	0.0024 (12)	0.0062 (12)	−0.0016 (12)
C3	0.0422 (17)	0.0358 (17)	0.063 (2)	−0.0028 (13)	0.0096 (15)	0.0017 (14)
C4	0.0454 (18)	0.0444 (19)	0.067 (2)	0.0018 (15)	0.0191 (16)	0.0139 (16)
C5	0.0493 (18)	0.0481 (18)	0.0387 (16)	0.0036 (14)	0.0138 (14)	0.0088 (13)
C6	0.0478 (17)	0.0414 (18)	0.0429 (17)	−0.0018 (14)	0.0063 (14)	−0.0012 (13)
I1	0.07890 (19)	0.07058 (18)	0.04761 (14)	−0.02011 (13)	0.01516 (12)	−0.01476 (11)
N1	0.0414 (13)	0.0395 (14)	0.0417 (14)	−0.0054 (11)	0.0055 (11)	0.0003 (11)
N2	0.0386 (13)	0.0388 (14)	0.0420 (13)	−0.0048 (11)	0.0040 (11)	0.0009 (11)
C7	0.0339 (14)	0.0373 (16)	0.0384 (15)	0.0009 (12)	0.0045 (12)	0.0029 (12)
C8	0.0377 (15)	0.0369 (16)	0.0393 (15)	0.0032 (12)	0.0051 (12)	0.0028 (12)
C9	0.0412 (16)	0.0395 (17)	0.0541 (19)	−0.0053 (13)	0.0050 (14)	−0.0001 (14)
C10	0.0509 (19)	0.049 (2)	0.056 (2)	−0.0044 (15)	0.0161 (16)	0.0109 (16)
C11	0.059 (2)	0.055 (2)	0.0338 (15)	−0.0093 (16)	0.0077 (14)	0.0050 (14)
C12	0.0529 (19)	0.0504 (19)	0.0393 (16)	−0.0092 (15)	0.0030 (14)	−0.0020 (14)
I2	0.07077 (17)	0.06949 (17)	0.04103 (13)	−0.01261 (12)	0.01232 (11)	−0.01094 (10)

### Geometric parameters (Å, º)

**Table:**

C1—C2	1.384 (4)	N2—C7	1.419 (3)
C2—C3	1.391 (4)	C7—C8	1.386 (4)
C3—C4	1.374 (5)	C8—C9	1.390 (4)
C4—C5	1.378 (5)	C9—C10	1.370 (4)
C5—C6	1.375 (4)	C10—C11	1.374 (5)
C6—C1	1.393 (4)	C11—C12	1.374 (4)
C2—I1	2.085 (3)	C12—C7	1.390 (4)
C3—H1	0.9300	C8—I2	2.086 (3)
C4—H2	0.9300	C9—H5	0.9300
C5—H3	0.9300	C10—H6	0.9300
C6—H4	0.9300	C11—H7	0.9300
C1—N1	1.423 (3)	C12—H8	0.9300
N1—N2	1.236 (3)		
			
C1—C2—C3	120.3 (3)	N1—N2—C7	115.1 (2)
C2—C3—C4	119.7 (3)	C7—C8—C9	120.3 (3)
C3—C4—C5	120.3 (3)	C8—C9—C10	119.6 (3)
C4—C5—C6	120.3 (3)	C9—C10—C11	120.4 (3)
C5—C6—C1	120.1 (3)	C10—C11—C12	120.4 (3)
C6—C1—C2	119.2 (3)	C11—C12—C7	120.1 (3)
C1—C2—I1	121.2 (2)	C12—C7—N2	123.5 (3)
C3—C2—I1	118.5 (2)	C8—C7—C12	119.1 (3)
C2—C3—H1	120.1	C8—C7—N2	117.4 (2)
C4—C3—H1	120.1	C7—C8—I2	120.6 (2)
C3—C4—H2	119.9	C9—C8—I2	119.1 (2)
C5—C4—H2	119.9	C8—C9—H5	120.2
C4—C5—H3	119.8	C10—C9—H5	120.2
C6—C5—H3	119.8	C9—C10—H6	119.8
C5—C6—H4	119.9	C11—C10—H6	119.8
C1—C6—H4	119.9	C10—C11—H7	119.8
C6—C1—N1	122.8 (3)	C12—C11—H7	119.8
C2—C1—N1	117.9 (3)	C11—C12—H8	119.9
C1—N1—N2	114.0 (2)	C7—C12—H8	119.9
